# Uropathogenic *E. coli* induces DNA damage in the bladder

**DOI:** 10.1371/journal.ppat.1009310

**Published:** 2021-02-25

**Authors:** Camille V. Chagneau, Clémence Massip, Nadège Bossuet-Greif, Christophe Fremez, Jean-Paul Motta, Ayaka Shima, Céline Besson, Pauline Le Faouder, Nicolas Cénac, Marie-Paule Roth, Hélène Coppin, Maxime Fontanié, Patricia Martin, Jean-Philippe Nougayrède, Eric Oswald

**Affiliations:** 1 IRSD, INSERM, Université de Toulouse, INRA, ENVT, UPS, Toulouse, France; 2 CHU Toulouse, Hôpital Purpan, Service de Bactériologie-Hygiène, Toulouse, France; 3 VibioSphen, Prologue Biotech, Labège, France; 4 MetaToulLipidomics Facility, INSERM UMR1048, Toulouse, France; University of Utah, UNITED STATES

## Abstract

Urinary tract infections (UTIs) are among the most common outpatient infections, with a lifetime incidence of around 60% in women. We analysed urine samples from 223 patients with community-acquired UTIs and report the presence of the cleavage product released during the synthesis of colibactin, a bacterial genotoxin, in 55 of the samples examined. Uropathogenic *Escherichia coli* strains isolated from these patients, as well as the archetypal *E*. *coli* strain UTI89, were found to produce colibactin. In a murine model of UTI, the machinery producing colibactin was expressed during the early hours of the infection, when intracellular bacterial communities form. We observed extensive DNA damage both in umbrella and bladder progenitor cells. To the best of our knowledge this is the first report of colibactin production in UTIs in humans and its genotoxicity in bladder cells.

## Author summary

Colibactin is a bacterial genotoxin suspected of being involved in cancer. So far, the focus has been on colorectal cancer following asymptomatic intestinal carriage of genotoxic *Escherichia coli*. Here, we show for the first time that this genotoxin is produced in humans, by detecting it in the urine of patients suffering from urinary tract infections. We subsequently show in a rodent UTI model the genotoxic activity of colibactin in the bladder, including in regenerative cells. These results raise the hypothesis of a role of UTI in bladder cancer, which could be an interesting perspective for future investigation.

## Introduction

Urinary tract infections (UTIs) are one of the most common bacterial infections, affecting approximately 150 million individuals each year [1]. UTIs occur most frequently in women, with more than 60% of females diagnosed with a UTI during their lifetime [2]. The severity of these infections ranges from asymptomatic bacteriuria and cystitis, *i*.*e*. infections localised to the bladder, to urosepsis, which can be fatal. Recurrences are very frequent, since approximately 30% of women experience a new UTI episode after resolution of the initial infection [2]. In addition to their consequences in terms of morbidity, mortality and associated economic and societal losses, UTIs are also a major reason for antibiotic treatments and thus strongly contribute to the global issue of antibiotic resistance. *Escherichia coli* strains, termed uropathogenic *E*. *coli* (UPEC) cause approximately 80% of all UTIs. These strains belong mainly to phylogroup B2 in western countries, which is increasingly present in the intestinal microbiota, the reservoir of UPEC [3,4]. UPEC strains produce a large number of virulence factors [5–7]. In particular, several toxins have long been associated with UPEC pathogenicity, such as α-hemolysin and CNF1 toxins [8,9]. More recently, a large proportion of UPEC strains which carry *pks* pathogenicity island encoding the genotoxin colibactin have been described [10–13].

**Fig 1:**
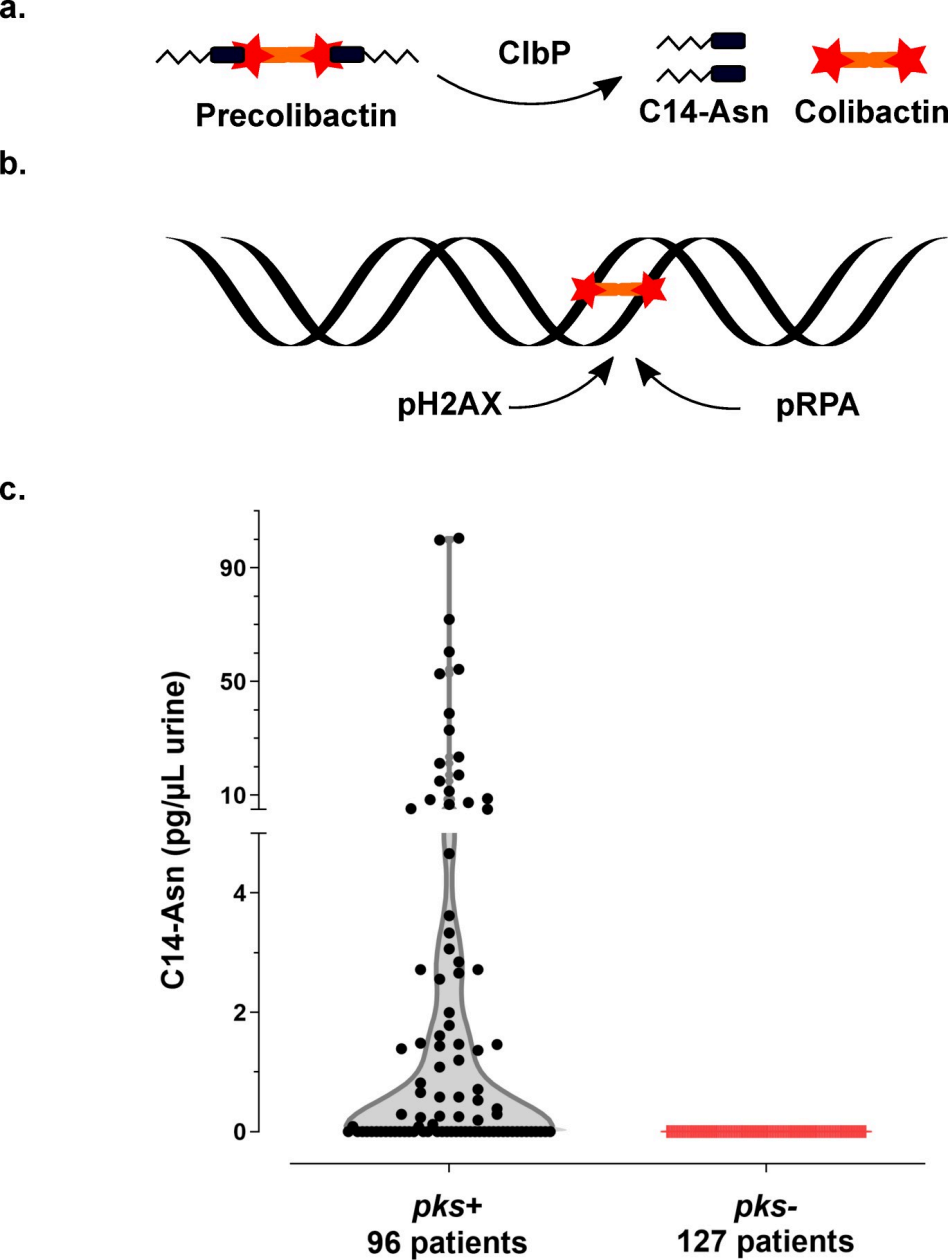
Synthesis of the genotoxin, colibactin, releases the C14-Asn metabolite detected in the urine of patients with UTI. **a.** Colibactin is first synthesised as a prodrug, precolibactin and then cleaved by ClbP peptidase thereby releasing the mature genotoxic dimeric colibactin, and C14-Asn. **b.** Colibactin alkylates both strands of the DNA helix, generating an interstrand cross-link. In response to this DNA damage, the host cell machinery recruits and phosphorylates the H2AX and RPA proteins. **c.** Concentration of C14-Asn in human urines according to the presence of a *pks* island in the genome of the corresponding UPEC isolate as determined by LC-MS/MS. All individual data points are shown on a violin plot, with a two segments linear Y range. C14-Asn was below the LC-MS/MS detection limit in the urine of patients infected with UPEC isolates that did not contain a *pks* island.

The *pks* pathogenicity island, composed of *clbA-S* genes, encodes a polyketide- non-ribosomal -peptide (PK-NRP) biosynthesis machinery [14]. Colibactin is first synthesised as an inactive prodrug by ClbN followed by the sequential interventions of multiple Clb enzymes. The ClbP peptidase subsequently cleaves the C14-Asparagine (C14-Asn) motif thereby releasing the mature, active form of colibactin with its twin warheads (Fig 1A) [15–17]. The genotoxin alkylates adenine residues on both strands of DNA, producing DNA interstrand cross-links [18–20]. These highly toxic DNA lesions initiate a DNA damage response, by phosphorylating replication protein A (pRPA) and phosphorylating the H2AX histone variant (pH2AX) (Fig 1B) [14,18]. Incomplete repair of this DNA damage can result in gene mutations [21]. *E*. *coli* strains carrying *pks* island have been shown to promote colon carcinogenesis in different mouse models [22–24]. In epidemiological studies, *pk*s+ *E*. *coli* strains are more prevalent in the gut microbiota of patients with colorectal cancer and a distinct mutational signature in human cancer genomes, predominantly colorectal tumours, was recently associated with colibactin genotoxic activity, further implicating an involvement of colibactin-producing *E*. *coli* in tumorigenesis [22,23,25,26]. This mutational signature has also been identified in tumours of the urinary tract [26].

Our current study shows that colibactin producing bacteria induce DNA damage in bladder cells, including in urothelial regenerative cells and that colibactin is produced by *pks*+ UPEC clinical strains isolated from human UTIs.

## Results

### Evidence of colibactin production in the urine of patients infected with UPEC

We collected urine samples from 223 adult patients with community-acquired pyelonephritis, cystitis or asymptomatic bacteriuria caused by *E*. *coli* at the University Hospital of Toulouse, France. Urine samples were analysed for the presence of C14-Asn, the aminolipid released during the final colibactin maturation step (Fig 1A). In contrast to the highly reactive and unstable colibactin, C14-Asn is stable and can be quantified by LC-MS/MS. C14-Asn was detected in urine samples of one quarter (55/223) of UTI patients, including asymptomatic infections (Fig 1C and S1 Table). We isolated *E*. *coli* strains harbouring genomic *pks* island from all urine samples which also contained C14-Asn. Conversely, C14-Asn was below the LC-MS/MS detection limits in urine samples of patients infected with *E*. *coli* strains which did not carry the *pks* pathogenicity island.

### Phylogenetic distribution of *pks* island in UPEC strains

**Fig 2:**
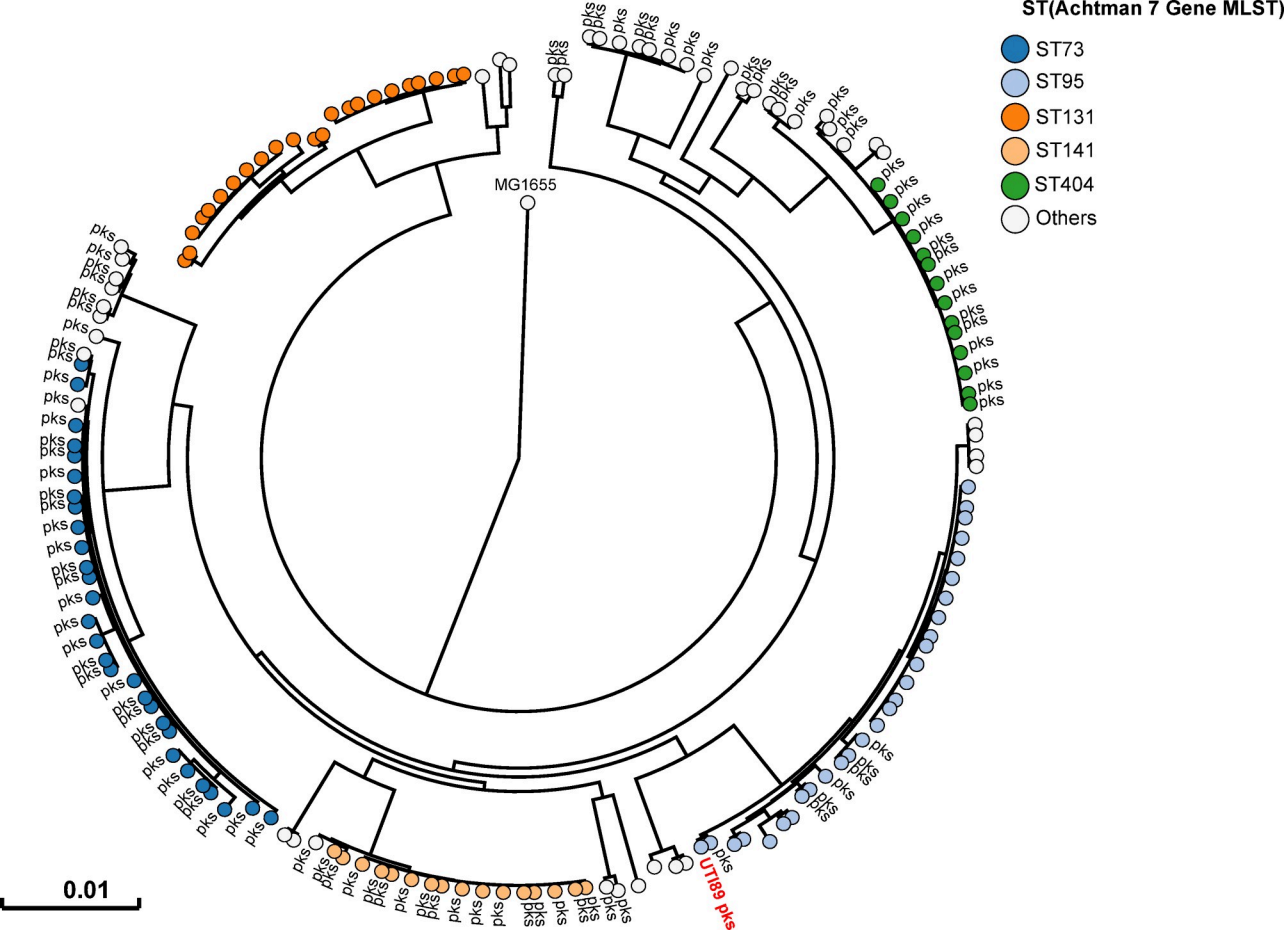
Human *pks*+ UPEC belong to major lineages of extraintestinal pathogenic *E*. *coli* from phylogroup B2. A phylogenetic tree based on whole genome analysis was constructed and rooted on *E*. *coli* MG1655. Each circle represents an individual UPEC strain. Main Sequence Types are grouped by colours. The presence of a *pks* island is denoted adjacent to each circle. The archetypal UPEC strain UTI89, which is *pks*+, was also included in this tree.

All 225 *E*. *coli* strains isolated during this sampling campaign were whole genome sequenced. The phylogenetic distribution of these *E*. *coli* isolates was typical of strains which cause UTIs [2]. A majority of strains belonged to the phylogroups B2 (69%) and D (15%) (Fig 2 and S1 Table). Forty three percent of strains harboured *pks* island. All the *pks*+ strains belonged to the phylogenetic group B2 and to the most common sequence types (STs) of extra-intestinal pathogenic *E*. *coli* such as ST73, ST95, ST141 and ST404 (Fig 2). As expected for extra-intestinal pathogenic *E*. *coli*, multiple known or suspected virulence genes were also present in the genome of these *pks*+ strains (S2 Table). *pks* island are thus widely present in the typical UPEC strains that cause community-acquired UTIs.

### UPEC strains carrying *pks* island produce the genotoxin colibactin

**Fig 3:**
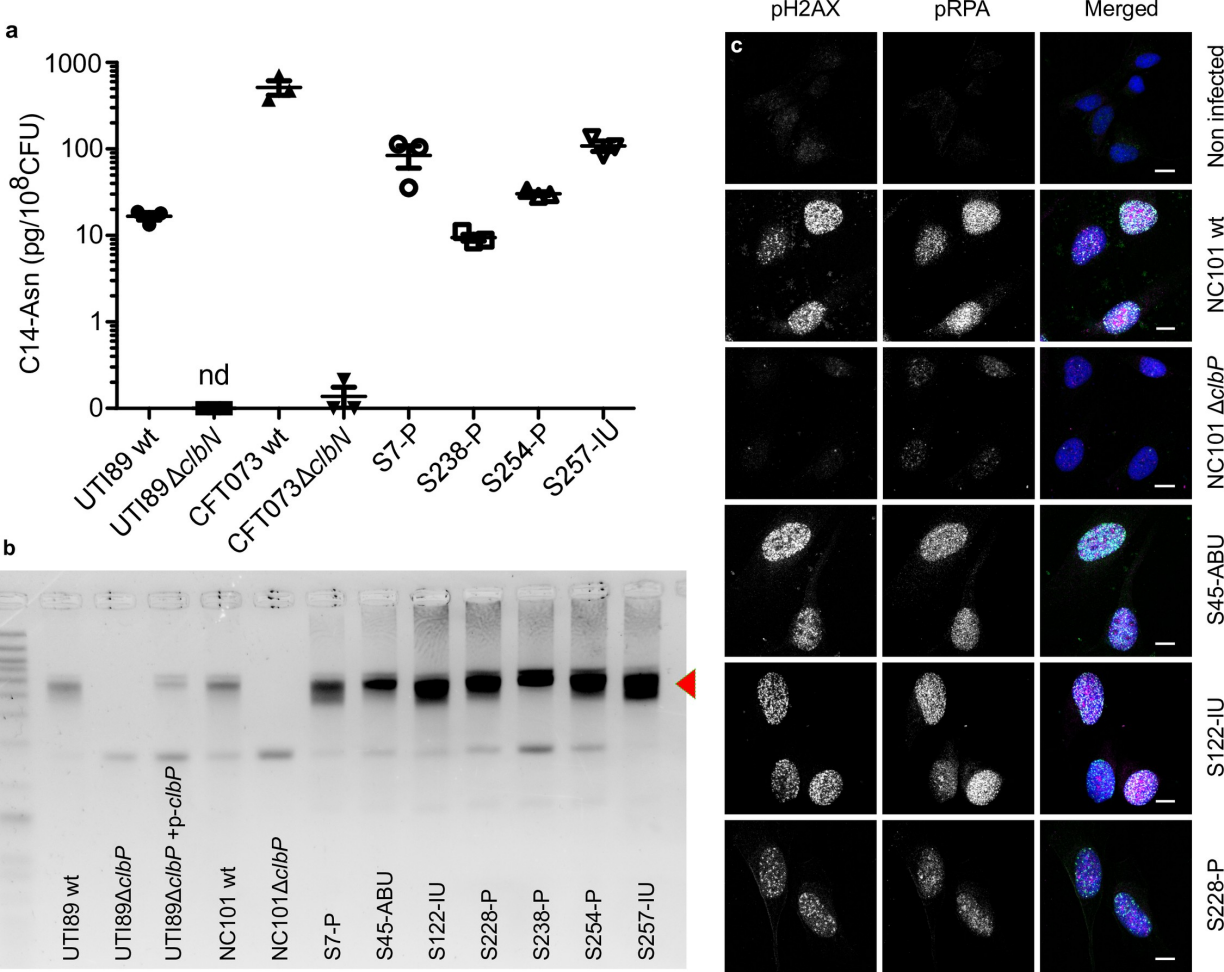
The archetypal cystitis *E*. *coli* strain UTI89 and clinical *pks*+ UPEC isolates produce colibactin and induce DNA damage. **a**. LC-MS/MS quantification of the C14-Asn cleavage product released following colibactin maturation by the ClbP peptidase in bacterial pellets of UPEC strains UTI89, CFT073 and their respective Δ*clbN* mutants (which are not able to synthesise C14-Asn), and four clinical UPEC strains S7, S238, S245, S257. Quantifications were performed in triplicate and represented as a mean ± standard error of the mean (SEM). nd: not detected **b.** Formation of DNA interstrand cross-links upon exposure of linear double stranded DNA to UTI89, NC101 and their respective Δ*clbP* mutants as well as seven representative clinical human UPEC isolates (S7, S45, S122, S228, S238, S254, S257), visualised by electrophoresis under denaturing conditions. DNA with interstrand cross-links (red arrow) migrates at an apparent molecular weight of twice that of DNA without cross-links. In the absence of any crosslinking DNA migrates as a single denatured strand. The gel shown is representative of three independent experiments. **c.** S33p-RPA32 and pH2AX immunofluorescence staining of HeLa cells, 16 hours after infection with the reference NC101 strain, its Δ*clbP* mutant or human UPEC strains S45, S122, S228. pH2AX and S33p-RPA32 = pRPA: grayscale. Merged: green = pH2AX; magenta = S33p-RPA32; blue = DAPI; scale bar = 10 μm. Human UPEC strains from AS = asymptomatic bacteriuria; IU = cystitis; P = pyelonephritis.

We next sought to confirm that *pks*+ UPEC strains expressed colibactin. We readily detected C14-Asn in *in vitro* cultures of both clinical UPEC isolates and the archetypal strains UTI89 (cystitis collection strain, commonly used in rodent models of UTI) and CFT073 (pyelonephritis collection strain) (Fig 3A). Production of C14-Asn depended on a fully functional colibactin assembly line, as mutation of the ClbN synthase gene abrogated its detection (Fig 3A). As for the strain NC101, which has been shown to be pro-carcinogenic in colorectal cancer mouse models, we detected formation of interstrand DNA crosslink by UTI89, but not by *clbP* mutants not able to maturate colibactin anymore (Fig 3B). The same DNA crosslinking effect was observed with clinical *pks+* UPEC isolates from asymptomatic bacteriuria, cystitis or pyelonephritis cases (Fig 3B). As was shown for NC101, infecting human epithelial cells with *pks+* UPEC strains induced the formation of nuclear pRPA and pH2AX foci, indicating that the DNA of exposed cell is damaged (Figs 1B and 3C). All together, these results demonstrate that UPEC strains *pks* island are functional, mediate the synthesis of colibactin and are genotoxic.

### Colibactin is produced during UTIs and induces bladder urothelium DNA damage

**Fig 4:**
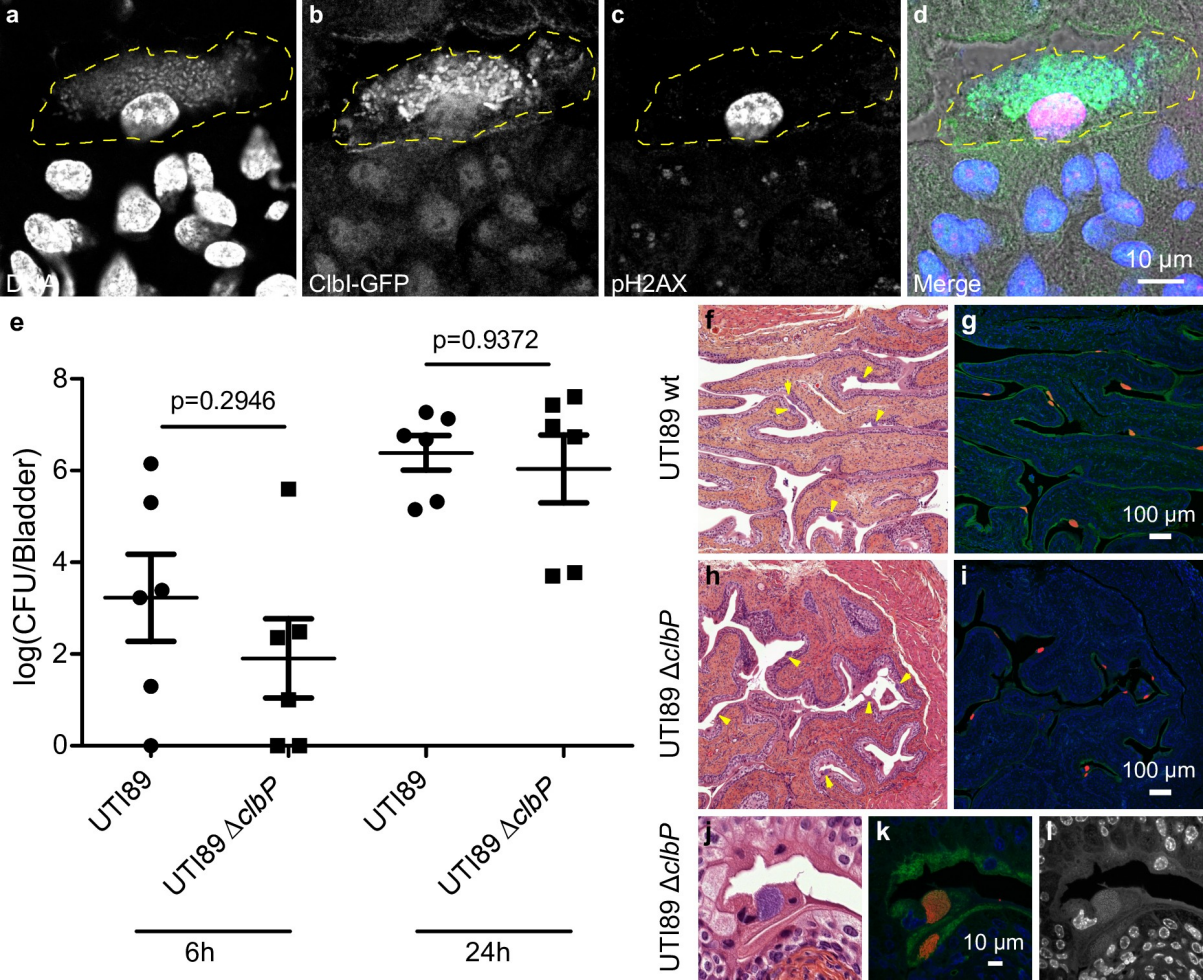
Colibactin is produced during UTI and induces DNA damage. **a-d**. Confocal microscopic detection of ClbI-GFP expression and pH2AX in frozen bladder sections, 6 hours post infection with UTI89, hosting the p-*clbI*-*gfp* fusion plasmid. The individual channel images are shown in grayscale, the umbrella cell containing the IBC is circled with a yellow dashed line. In the merged (d) image: blue = DNA, green = ClbI-GFP, magenta = pH2AX, grey = phase contrast. The immunofluorescence staining was repeated three times on different slides, other representative images are shown in S1 Fig. **e.** Bladder tissue *E*. *coli* counts, 6 h and 24 h after infection with wild-type *E*. *coli* UTI89 (circles) or Δ*clbP* (squares). Each data point corresponds to one mouse (n = 6), with mean ± standard error of the mean (SEM) shown for each group. Mann-Whitney U test. **f-l.** Bladder sections 6 hours post inoculation with wild-type UTI89 (f, g) or the isogenic Δ*clbP* mutant (h-l) stained with haematoxylin-eosin (f,h,j), FISH (g,i,k) or DAPI (l, grayscale) all of which detect IBCs (arrows). In the FISH images: blue = DAPI stain DNA; green = FITC-conjugated wheat germ agglutinin (WGA) stained polysaccharides; red = bacterial cells, labelled with the universal 16S FISH probe. The images are representative of the 5 animals per group observed.

In order to monitor the expression of the *pks* synthesis machinery during a UTI, we transformed the UPEC strain UTI89 with a plasmid expressing a GFP tagged *pks* island-encoded polyketide ClbI synthase from its endogenous promoter. In bladder tissue collected 6 hours after infection, we observed ClbI-GFP expressing bacteria in intracellular bacterial communities (IBCs) inside superficial umbrella cells which line the lumen of the bladder (Fig 4A–4D and S1). Production of colibactin was further confirmed by the detection of C14-Asn in pooled urines from 8 mice 24 hours after infection (C = 0.66 pg/μL). We next assessed whether the metabolically active *pks* machinery was associated with DNA damage in bladder cells. Phosphorylation of H2AX was readily detected in nuclei of umbrella cells containing IBCs, 6 hours after infection with wild-type *E*. *coli* UTI89 with or without the p-*clbI*-*gfp* plasmid (Fig 4C and 4D and S1 and S2). A *clbP* mutant unable to produce colibactin was very weakly genotoxic or not genotoxic at all (S2 Fig), although equally capable of colonising the bladder (Fig 4E) and inducing the formation of IBCs (Fig 4F–4L). These results show that colibactin is expressed *in vivo*, in the bladder from the very early stages of UTI and can induce DNA damage.

### Colibactin induces urothelial cell DNA damage in the regenerative compartment

**Fig 5:**
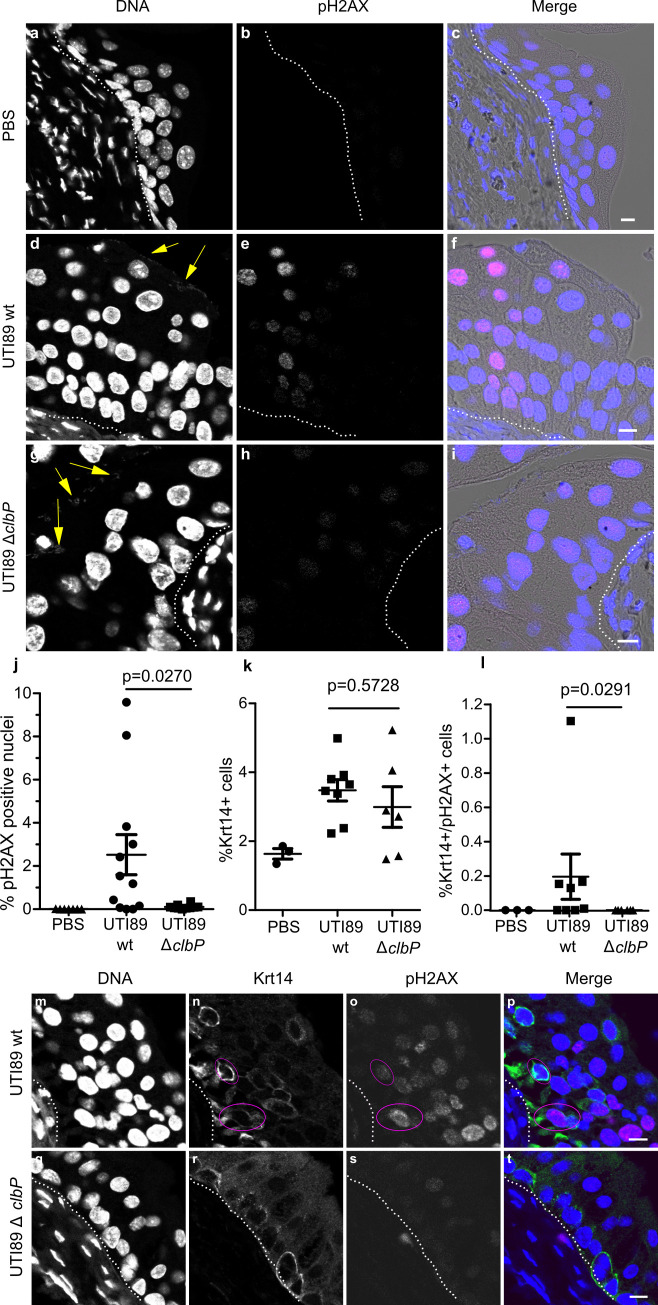
Colibactin induces DNA damage in urothelial cells of the regenerative compartment. **a-i.** Immunofluorescence staining of pH2AX on paraffin-embedded bladder sections 24 hours after PBS inoculation (a-c), or UTI89 wild-type (d-f) or UTI89Δ*clbP* (g-i) infection. The individual fluorescence channel images are shown in grayscale. Merged images: blue = DNA, magenta = pH2AX, grey = phase contrast. Yellow arrows highlight bacteria, white dotted lines represent the basal membrane of the urothelium. Scale bar = 10 μm. The experiment was repeated twice on 5 to 8 mice, other representative images are shown in S3 Fig. **j-l.** pH2AX positive nuclei (j), Krt14 positive cells (k) and Krt14 and pH2AX positive cells (l) were counted objectively using Fiji and CellProfiler softwares on the confocal images from large mosaic of bladder sections. Each dot represents one mouse bladder. Mann Whitney U-test performed between UTI89 wild-type and UTI89 Δ*clbP*. **m-t.** Paraffin-embedded bladders sections were immuno-stained for pH2AX and Krt14 24 hours post infection with UTI89 wild-type (m-p) or UTI89Δ*clbP* (q-t). The individual fluorescence channel images are shown in grayscale. Merged images: blue = DNA, magenta = pH2AX, green = Krt14. Cells positive for both Krt14 and pH2AX are circled in pink. White dotted lines highlight the basal membrane of the urothelium. Scale bar = 10 μm. Other representative images are shown in S6 Fig.

The above results show that colibactin expression occurs as soon as 6 hours after infection, with DNA damage mainly in the nuclei of superficial cells. It is well known from mouse models with UTI89 that these superficial cells, especially those containing IBCs, are exfoliated within 6 to 12 hours [27]. The regeneration of the urothelium with a phase of intense proliferation of intermediate and basal cells occurs 24 hours after infection [27]. In order to study the consequences in terms of genotoxicity for the remaining urothelium of an infection by a colibactin-producing strain, we observed pH2AX in bladder sections 24 hours after wild-type *E*. *coli* UTI89 infection. We found significant numbers of positive pH2AX nuclei within the urothelium, generally grouped in patches, including cells from the bottom to the top of the urothelium (Fig 5D–5F). Bladders from mice infected with the *clbP* mutant did not exhibit such pH2AX positive cells pattern (Fig 5G–5I) and quantification of the number of positive pH2AX nuclei was significantly lower than in individuals infected with the wild-type strain (Fig 5J). pH2AX positive cells nevertheless reappeared after complementation with the wild-type *clbP* allele (S3 Fig). The DNA damage was not related to apoptosis as pH2AX+ urothelial cells were not detected with TUNEL apoptosis staining (S4 Fig).

The basal urothelium compartment harbours keratin-14 positive (Krt14+) progenitor cells, which are important for urothelium renewal following injury [28]. There was an increase of Krt14+ cells in urothelial tissue infected with either wild-type or *clbP* mutant UTI89 24 hours after infection (Figs 5K and S5). Interestingly, bladders infected with the genotoxic wild-type UTI89 strain exhibited low but significant numbers of urothelial cells that were positive for both the regenerative cell marker Krt14 and the DNA damage marker pH2AX (Fig 5L–5T). These cells were not positive for Uroplakin, a marker of intermediate and superficial cells (S7 Fig). Thus, during UTI with a *pks+* UPEC, colibactin induces DNA damage in superficial and basal regenerative urothelial cells.

## Discussion

It is now increasingly clear that *pks*+ *E*. *coli* strains found in the intestinal microbiota may play a role in the aetiology and pathogenesis of colorectal cancer. These *E*. *coli* strains of phylogenetic group B2 are often the same ones responsible for UTIs, but to date, no study has been conducted on colibactin and UTIs. To the best of our knowledge the current study is the first to report the presence of a C14-Asn signature, a metabolite of colibactin production, in the urine of patients infected with *pks*+ UPEC. Thus, colibactin is produced during UTIs, one of the most common human bacterial infections worldwide. We used a mouse model of human UTI, to demonstrate that colibactin is produced and that it induces DNA damage in urothelial cells, including in some (low but significant numbers) bladder Krt14+ progenitor cells.

Our study confirms the frequent occurrence of *pks*+ UPEC observed in Europe and the US, irrespective of infection severity [10–13]. This high prevalence in human subjects is in contrast to the apparent lack of selective advantages associated with the production of colibactin in UTIs mouse models. Our results suggest that colibactin is not essential in our mouse model for the ability of UPEC to colonise the bladder or to form IBCs, whereas colibactin has been shown to play a role in the pathogenicity of *E*. *coli* in extraintestinal infections such as septicemia or meningitis [29,30]. However, it should be noted that the standard mouse model we used omits a key step in the pathogenesis of UTIs: the domination and emergence from the intestinal reservoir [31]. *E*. *coli* strains carrying *pks* island are commonly observed among strains with a greater ability to persist in the intestinal microbiota [3]. In addition to the potential role of colibactin in modulating the intestinal microbiota and promoting gut colonisation, enzymes encoded by *pks* island are essential for the synthesis of siderophores and siderophore-microcins [32,33]. Siderophores are major determinants in the domination of the intestinal niche [34]. They confer upon strains that produce them the ability to outcompete other bacteria for iron, a rare essential nutrient. Moreover, siderophore-microcins, antimicrobial peptides which target phylogenetically-linked enterobacteria help UPEC to implant and proliferate within their intestinal reservoir, a step which precedes UTI [10].

The role of *pks* island in UPEC cannot merely be reduced to a determinant of intestinal colonisation. Indeed, our current study shows that the *pks* machinery is also active in the urinary tract and even within IBCs. Bacterial multiplication in these structures is intense, with a doubling time of nearly 30 min, which requires an efficient and optimised bacterial metabolism [35]. Although colibactin is a small molecule, the metabolic cost of its production, *i*.*e*. expressing and operating the PK-NRP biosynthesis machinery is very high: nearly 1000 times higher than that of peptide synthesis [36]. As UPEC trigger such energetically inexpedient assembly lines during UTIs, one may speculate that they must derive an adaptive benefit from it. Other products of the machinery may possibly confer this advantage. Indeed, there is a wide diversity of metabolites produced by the *pks* machinery (at least *in vitro)*, ranging from other putative forms of “colibactins” (such as macrocyclic metabolites) to other smaller metabolites, which potentially vary between strains [15,37]. The biological function of these metabolites remains to be elucidated, but some of these metabolites may be relevant to the pathogenesis of infections. We have, for instance, recently described the synthesis of C12-Asn-GABA, by the *pks* machinery of the probiotic Nissle 1917 strain and shown its digestive pain-relieving activity [38]. The production of such an analgesic metabolite coupled with the increased production of siderophores by *pks*+ *E*. *coli* strains may provide a selective advantage to colonising the urinary tract, in addition to the digestive tract. Future experiments should aim at examining the impact of the various *pks*-associated factors on the course of the infection.

Irrespective of the role of *pks* island in the virulence of UPECs, the fact remains that the archetypical UPEC strain UTI89 is experimentally genotoxic to mouse bladder cells. Colibactin-induced DNA damage can be incompletely repaired, resulting in the accumulation of gene mutations and cell transformation [21,26,39]. We have previously shown that post-exposure chromosomal instability to colibactin can persist in daughter cells [21]. DNA damage could also be propagated to contiguous cells by the induction of a senescence-associated secretory phenotype in the affected cells and the production of ROS, that could also explain the pattern of pH2AX positive patches of cells 24 hours after infection [39,40]. In the current study, we observed that some murine bladder progenitor cells (Krt14+) display pH2AX positive nuclei. One could speculate about the long-term consequences of such genotoxic stress on the urothelium, especially for patients with recurrent infections. In humans, Krt14 expression characterises undifferentiated bladder cancers with particularly poor prognoses [41,42]. Rodent bladder cancer models indicate that a significant proportion of cancerous tissue derives from Krt14 positive cells [28]. Thus, we could hypothesise that colibactin damage to Krt14+ cells may initiate and propagate DNA lesions. In line with this hypothesis, the colibactin mutational signature has recently been identified in colorectal cancers but also in urinary tract cancers [26,43]. Currently the main risk factors for bladder cancers are tobacco and occupational exposure to solvents, which are more frequently investigated than UTIs [44]. However, a large worldwide bladder cancer case control study recently showed that regular UTIs were epidemiologically associated with an increased risk of urinary bladder cancer [45]. We thus suggest that *pks*+ UPEC should be now studied as a possible additional risk factor, particularly in cases of chronic and regular infections, irrespective of whether symptoms are present or not. We detected C14-Asn in the urine of patients with asymptomatic bacteriuria, which are usually not treated with antibiotics and can thus persist for periods of up to many years [46]. A better understanding of the consequences of colibactin production could prompt a systematic search for *pks* island in UPEC isolates or C14-Asn in the urine of patients at risk.

## Materials and methods

### Ethics statement

According to the French regulations relating to observational database analyses, the collection of clinical *E*. *coli* strains and urine samples did not require specific informed consent. The data were analysed anonymously.

Mouse UTI experiments were performed in accordance with the European directives for the protection of animals used for scientific purposes (2010/63/EU). The local ethics committee from CREFRE-US006 (Regional Centre for Functional Exploration and Experimental Resources) approved the protocol (number CEEA-122 2014–53).

### Bacterial strains

The archetypal *E*. *coli* strains used in this study were the UPEC strains UTI89 [47], CFT073 [9] and the colitogenic *E*. *coli* strain NC101 [22]. The Δ*clbP* and Δ*clbN* mutants were constructed using the lambda Red recombinase method [48] with primers IHAPJPN29 and IHAPJPN30 for Δ*clbP* [14] and primers clbN-P1 and clbN-P2 for Δ*clbN* [38]. The pMB702 construct (referred to as p-*clbP*) was used for complementation of the UTI89 Δ*clbP* mutant [49]. For the *in vivo* complementation assay, the UTI89 Δ*clbP* mutant was transformed with the pCM17-*clbP* plasmid. Briefly, the *clbP* gene was PCR-amplified from pBRSK-*clbP* [49], with primers clbP-F-Bam_pm (5’-atGGATCCatgacaataatggaacacgttagc-3’) and pBRSK-F-Bam_pm (5’-atGGATCCcaagctcgga attaaccctc-3’) and cloned into the pCM17 vector [50] BamHI site. The ClbI C-terminal GFP fusion was constructed using the Gibson Assembly kit (New England Biolabs, MA, USA). Briefly, the *clbI* and *gfp* genes were amplified by PCR using the following primers: pK184-clbI-gb1(5’-GATTACGAATTCGAGCTCGGTACCCATGGCAGAGAATGATTTTGG-3’); clbI-gfp-gb2 (5’-CTTCTCCTTTTCCGCCTCCTCCGCCCTCATTAATCATGTCGTTAACTAG-3’); clbI-gfp-gb3 (5’-GATTAATGAGGGCGGAGGAGGCGGAAAAGGAGAAGAACTTTTCACTGG-3’) and gfp-pK184-gb4 (5’-TGCAGGTCGACCTCGAGGGATCCCCTTATTTGTATAGTTCATCCATGCC-3’). A glycine linker (5’-GGCGGAGGAGGCGGA-3’) was introduced at the *clbI* and *gfp* junction for flexibility. PCR amplified fragments and SmaI-digested pK184 vector were assembled according to the manufacturer’s recommendations.

### Collection of clinical strains and urines

The collection of 225 *E*. *coli* strains from urine samples at the Adult Emergency Department of Toulouse University Hospital, France, between July and October 2017, was previously described [10]. Urine was collected from 223 patients (women and men, under 75 years) with either pyelonephritis (104), symptomatic infections excluding pyelonephritis (cystitis) (83), or asymptomatic bacteriuria (36) without urological comorbidities or catheterisation. All strains were identified as *E*. *coli* by matrix-assisted laser desorption/ionisation time-of-flight mass spectrometry (Microflex LT MALDI-TOF MS, Bruker Daltonik GmbH, Germany). Samples of the corresponding urine collected in boric acid tubes were stored at -80°C until lipid analysis.

### C14-Asn quantification

Extraction and quantification of C14-Asn was performed as previously described [38]. Briefly, 5 μL internal standard mixture (Deuterium-labelled compound at 400 ng/mL) was added to the bacterial pellets of 24 h DMEM cultures or to 500 μL of urine samples before crushing, followed by addition of cold methanol (MeOH) (15% final volume) and homogenisation. After centrifugation, supernatants were solid phase extracted on HLB plates (OASIS HLB 2 mg, 96-well plate, Waters, Ireland). Following washing with H_2_O/ MeOH (90:10, v/v) and elution with MeOH, samples were evaporated twice under N_2_ and finally resuspended in 10 μL MeOH. Separation and quantification of C14-Asn was performed on a high-performance liquid chromatography coupled to tandem mass spectrometry system (G6460 Agilent) [38]. Limit of detection was 2.5 pg and limit of quantification was 5 pg.

### Sequencing data, sequence alignments and phylogenetic analyses

Whole genome sequencing was performed using the Illumina NextSeq500 Mid Output platform (Integragen, Evry, France) to generate 2 x 150 bp paired-end reads, at approximately 80x average coverage. Genome *de novo* assembly and analysis were performed with the BioNumerics 7.6 software (Applied Maths) and Enterobase (http://enterobase.warwick.ac.uk/). For SNP-based phylogenetic trees, core genome alignments were generated after mapping raw reads to the *E*. *coli* MG1655 genome. The core genome phylogenetic tree was inferred with the Maximum-likelihood algorithm using Enterobase for B2 phylogroup strains. Sequences of all isolates are available in the NCBI Database, Bioproject number PRJNA615384 (https://www.ncbi.nlm.nih.gov/bioproject/PRJNA615384).

### Exogenous DNA cross-linking assay

As previously described [18], the pUC19 plasmid, linearised with BamHI, was exposed to bacteria pre-grown (3 x 10^6^ CFU) in DMEM 25 mM Hepes for 40 min at 37°C. The DNA was purified and 100 ng submitted to denaturing gel DNA electrophoresis (40 mM NaOH and 1 mM EDTA, pH ~12.0). After a neutralisation step, the gel was stained with GelRed and visualised under UV using the ChemiDoc Imaging System (BioRad).

### pH2AX and pRPA immunofluorescence analysis of post infected HeLa cells

HeLa cells (ATCC CCL2) were cultured and infected in 8-well chamber slides (Labtek), as previously described [18]. 24 hours after passaging, Hela cells were infected with bacteria precultured in DMEM 25 mM HEPES at a multiplicity of infection (MOI) of 50, for 4 h. Cells were then washed and incubated overnight in cell culture medium with 100 μg/ml gentamicin. Immunofluorescence analysis was then performed as previously described [18]. Briefly, cells were pre-extracted in PBS 0.1%, Triton X-100 and fixed in PBS 4% formaldehyde, permeabilised, blocked with MAXblock medium (Active Motif) and stained with antibodies against pH2AX (1:500, JBW301, Millipore) and S33p-RPA32 (1:500, A300-264A, Bethyl), diluted in MAXblock 0.05%, Triton X-100. Cells were washed 3 times for 5 min in PBS 0.05%, Triton X-100 and incubated with secondary AlexaFluor 488 or 568 (Invitrogen) diluted 1:500 in MAXblock medium with 1 μg/ml DAPI (Sigma). Slides were mounted with Fluoroshield (Sigma) and examined on a Leica SP8 laser scanning confocal microscope in sequential mode with LasX software (Leica), while keeping the same laser and detector settings between different wells. Final images were processed with the ImageJ software.

### Mouse UTI model

Female, 6–8 weeks old, C3H/HeN mice (Janvier Labs) were infected transurethrally as previously described [51–53]. Briefly, UPEC were cultivated statically in LB [51] and resuspended to an inoculum of 10^8^ CFU in 50 μl PBS. Mice under 4% isoflurane anaesthesia were inoculated twice at one-hour intervals with a pump that delivered 10 μL/s. At 6 or 24 hours, mice were euthanised by cervical dislocation (properly performed by experienced personnel), bladders were harvested, homogenised for bacterial enumeration on agar plates, stored in OCT compound at -80°C or fixed in 4% formaldehyde after filling the bladder prior to paraffin embedding. Each experiment was conducted in duplicate with 5 to 8 mice per group.

### Histological and immunofluorescence analyses of mouse bladder tissue

Histological bladder slices were prepared using standard protocols and processed for haematoxylin-eosin staining then scanned using a Pannoramic 250 scanner (3DHistech). Final images were captured using CaseViewer software (3DHistech).

For pH2AX, Krt14 and Uroplakin (Upk) staining on paraffin-embedded bladders, 5 μm thick sections were de-paraffinised and re-hydrated in xylene, ethanol and tap water. Unmasking was performed with a 6-min trypsin digestion (Trypsin-EDTA solution, Sigma, T392) followed by 30 min incubation in citrate buffer pH 6 at 80–95°C. Sections were then blocked and permeabilised in MaxBlock, 0.3% Triton X100. Slices were stained with anti-pH2AX (Rabbit, Cell Signalling S9718; or Mouse, Millipore #O5-636) at a dilution of 1:200, anti-Krt14 antibodies (Chicken, Ozyme BLE906001) at a dilution of 1:250 and anti-Upk III (Rabbit, Abcam, ab157801) at a dilution of 1:800 in MaxBlock, 0.3% Triton X100. Sections were washed in PBS, 0.05% Triton X100 and incubated with secondary antibodies (Alexa 633 Goat anti-Rabbit antibody, Invitrogen A21071, at a dilution of 1:200 or 1:500; Alexa 555 Goat anti-Chicken antibody, Invitrogen A32932, at a dilution of 1:500; Tetramethylrhodamine Goat anti-Mouse antibody, Invitrogen T2762, at a dilution of 1:200). Images were acquired as with infected cultured cells.

For demonstration of ClbI-GFP reporter expression, to achieve better GFP preservation, we used OCT compound-embedded bladders. Eight μm thick sections were dried on Superfrost plus glass slides, fixed for 15 min in 4% formaldehyde then rinsed with PBS, blocked and permeabilised with MaxBlock, 0.3% Triton X100. Primary anti-GFP (Rabbit, Abcam 6556) and anti-pH2AX (Mouse, Millipore #O5-636) antibodies were diluted at 1:200 in MaxBlock, 0.3% triton X100. After washing in PBS, 0.05% Triton X100, slides were incubated with secondary antibodies at 1:200 (Alexa 633 Goat anti-Rabbit antibody (Invitrogen A21071) and Alexa 488 Goat anti-Mouse antibody (Life technologies A11029)) and DAPI.

For pH2AX positive nuclei quantification, the Fiji software was used [54]. Briefly, images were analysed in batch with a macro that run a contrast enhancement (saturation = 0.35) and gaussian blur attenuation (sigma = 2) on the DAPI channel. To create a mask on this channel, “Auto threshold” with Otsu dark was used with holes filling and watershed. The “Analyse particles” command was then used to quantify the pH2AX signal within the nucleus region. For each batch of staining and acquisition, nuclei were counted positive when exhibiting a signal greater than 10 times the average signal on sections of the PBS mouse control analysed in parallel.

For Krt14 positive and Krt14/pH2AX positive cells quantification, the CellProfiler software was used [55]. Briefly, images were analysed in batch with a pipeline that first identify nuclei as primary objects with Otsu thresholding. Cells were defined by 6 pixels expansion from nuclei with “Distance-N” method and then cytoplasm were deducted by subtraction. pH2AX intensity in nuclei and Krt14 intensity in cytoplasm were measured and recorded for each cell. pH2AX positive cells were defined like after Fiji analysis. Krt14 positive cells were counted when exhibiting a signal above 3 times the average signal on the whole bladder section on the mosaic confocal image.

For TUNEL-staining of paraffin-embedded bladders, 5 μm thick sections were de-paraffinised and re-hydrated in xylene, ethanol and tap water. Sections were then treated by Proteinase K (15 μg/ml) in 10mM Tris/HCl (pH 7.8) at 37°C for 30 min and then rinsed twice in PBS, as previously already described for paraffin-embedded bladders [56]. As a positive control, slices from PBS inoculated animals were treated with DNAse I (2000 UI in 50 mM Tris-HCl, pH 7.5, 1 mg/ml BSA) for 10 min at room temperature. Slices were then TUNEL-stained using the In Situ Cell Death Detection Kit, TMR red (Roche) according to the manufacturer’s recommendations. Slices were mounted with Fluoroshield containing DAPI and images were acquired as with infected cultured cells.

### Fluorescence *in situ* hybridisation (FISH) of mouse bladder tissue

Five micron paraffin-embedded bladder sections were de-paraffinised in xylene/ethanol. Sections were incubated in lysozyme solution (10 mg/ml, Sigma, France) for 15 minutes at 37°C and exposed to 100 μl of universal bacterial 16 S fluorescent rRNA probe (Eub338, GCTGCCTCCCGTAGGAGT-Cy5’, Eurofins, France) at a concentration of 5 ng/μl, in hybridisation buffer (20 mM Tris-HCl, pH7.4, 0.9 M NaCl, 0.01% SDS) at 46°C for 3 hours [57]. Sections were then incubated in a 48°C prewarmed saline-sodium citrate wash buffer (30 mM sodium citrate, 300 mM sodium chloride, pH7.4, Invitrogen, France) for 20 minutes. To stain polysaccharide-rich content, sections were counterstained with FITC-conjugated wheat germ agglutinin (Invitrogen, France) at a 1:1000 dilution in PBS buffer for 30 min. Slides were mounted with Fluoroshield containing DAPI. Images were acquired using a confocal laser scanning microscope (Zeiss LSM 710) and final images were processed and edited with the ImageJ software.

### Statistical analyses

Graphical representation and statistical analyses were carried out using GraphPad Prism 8.3. P values were calculated using two-tailed Mann-Whitney U test.

## Supporting information

S1 Fig Immunofluorescence staining of GFP and pH2AX on frozen bladder sections 6 hours post infection with UTI89 p-*clbI*-GFP (a-l) and wild-type UTI89, non-expressing GFP (m-p). The individual channel images are shown in grayscale. In the merge images: blue = DNA, green = ClbI-GFP or GFP, magenta = pH2AX, grey = phase contrast. Umbrella cells containing an IBC are circled with a yellow dashed line, the basal side of the urothelium is delimited with a black dotted line. The images are representative of immunofluorescence staining repeated three times on different slides.
(TIF)

S2 Fig Colibactin induces DNA damage in umbrella cells containing IBCs at early stages of infection. Immunofluorescence staining of pH2AX on paraffin-embedded bladders sections 6 hours post-infection with the wild-type UTI89 (a, b) or the Δ*clbP* isogenic mutant (c,d). The individual channel images are shown in grayscale. Umbrella cells containing IBCs are circled with yellow dashed line and urothelium is delimited with green dotted line.
(TIF)

S3 Fig Colibactin induces DNA damage in urothelial cells. DAPI (DNA: a, d) and immunofluorescence staining of pH2AX (b, e) on paraffin-embedded bladders sections 24 hours post infection by UTI89Δ*clbP* (a-c) or UTI89Δ*clbP*+pCM17*clbP* (d-f). For immunofluorescence, the individual channel images are shown in grayscale. Merged images: blue = DNA, magenta = pH2AX, grey = phase contrast.
(TIF)

S4 Fig pH2AX positive cells are not apoptotic cells. TUNEL staining of paraffine-embedded bladder sections 24 hours post PBS inoculation (a-f) or wild-type UTI89 infection (g-j; k-n) in C3H/HeN mice. Sections were treated with DNAse I as a TUNEL positive control (d-f). TUNEL staining of infected bladder sections was compared to pH2AX immunofluorescence on a serial section (j is a section next to g-i and n is next to k-m). The individual fluorescence channel images are shown in grayscale. Merged images: blue = DNA; red = TUNEL; green = pH2AX. Yellow dotted lines represent the basal membrane of the urothelium. Scale bar = 100 μm.
(TIF)

S5 Fig Increase Krt14 positive cell numbers upon UTI. Immunofluorescence staining of Krt14 and DAPI stained DNA on paraffin-embedded bladders sections 24 hours post PBS inoculation (a-b) or infection by UTI89 wild-type (c-d) or UTI89 Δ*clbP* (e-f). The individual channel images are shown in grayscale. Pink arrows: Areas with Krt14+ cells. Urothelium is delimited with green dotted line. Scale bar = 100 μm. Quantification of Krt14 cells is shown in Fig 5K.
(TIF)

S6 Fig Colibactin induces DNA damage in urothelial cells of the regenerative compartment. Paraffin-embedded bladders sections were immuno-stained for pH2AX and Krt14 24 hours post infection with UTI89 wild-type (a-d) or UTI89 Δ*clbP* (e-h). The individual fluorescence channel images are shown in grayscale. Merged images: blue = DNA, magenta = pH2AX, green = Krt14. Cells positive for both Krt14 and pH2AX are circled in pink. Scale bar = 10 μm.
(TIF)

S7 Fig Colibactin induces DNA damage in basal Uroplakin-negative cells. a-c. Immunofluorescence staining of Upk and Krt14 on paraffin-embedded bladder sections 24 hours after UTI89 wild-type infection. d-f. Immunofluorescence staining of Upk and pH2AX on a serial section next to the one shown in a-c. Cells positive for pH2AX but negative for Upk are circled with yellow dashed line. The individual fluorescence channel images are shown in grayscale. Merged images: green = Upk, red = Krt14, magenta = pH2AX, blue = DNA. White dotted lines represent the basal membrane of the urothelium. Scale bar = 10 μm. f. Insert: higher magnification: green = Upk, magenta = pH2AX, gray = contrast.
(TIF)

S1 Table Human *pks*+ UPEC belong to major lineages of phylogroup B2 ExPEC. Phylogroup, Sequence Types and presence of a *pks* island were determined from whole genome sequences of UPEC isolates, along with the detection of C14-Asn from the corresponding urine.
(DOCX)

S2 Table Characterisation of UPEC strains isolated. (XLSX)
