# Supplementary figures and images for: Uropathogenic E. coli induces DNA damage in the bladder
Source: PLoS Pathog. 2021 Feb 25;17(2):e1009310. doi: 10.1371/journal.ppat.1009310 (PMC7906301; doi:10.1371/journal.ppat.1009310)

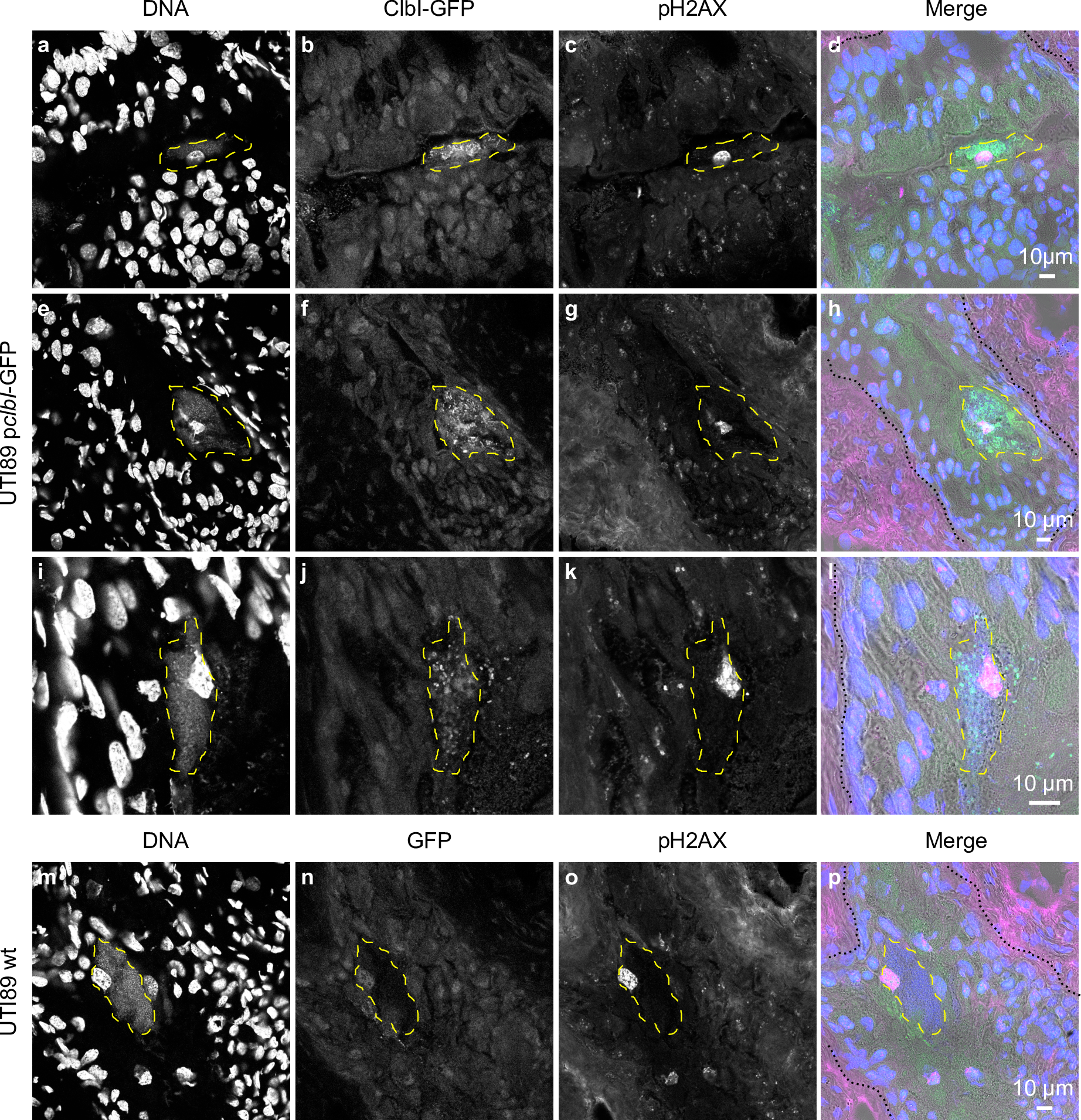

Supplement: S1 Fig — The individual channel images are shown in grayscale. In the merge images: blue = DNA, green = ClbI-GFP or GFP, magenta = pH2AX, grey = phase contrast. Umbrella cells containing an IBC are circled with a yellow dashed line, the basal side of the urothelium is delimited with a black dotted line. The images are representative of immunofluorescence staining repeated three times on different slides. (TIF) [file ppat.1009310.s001.tif]

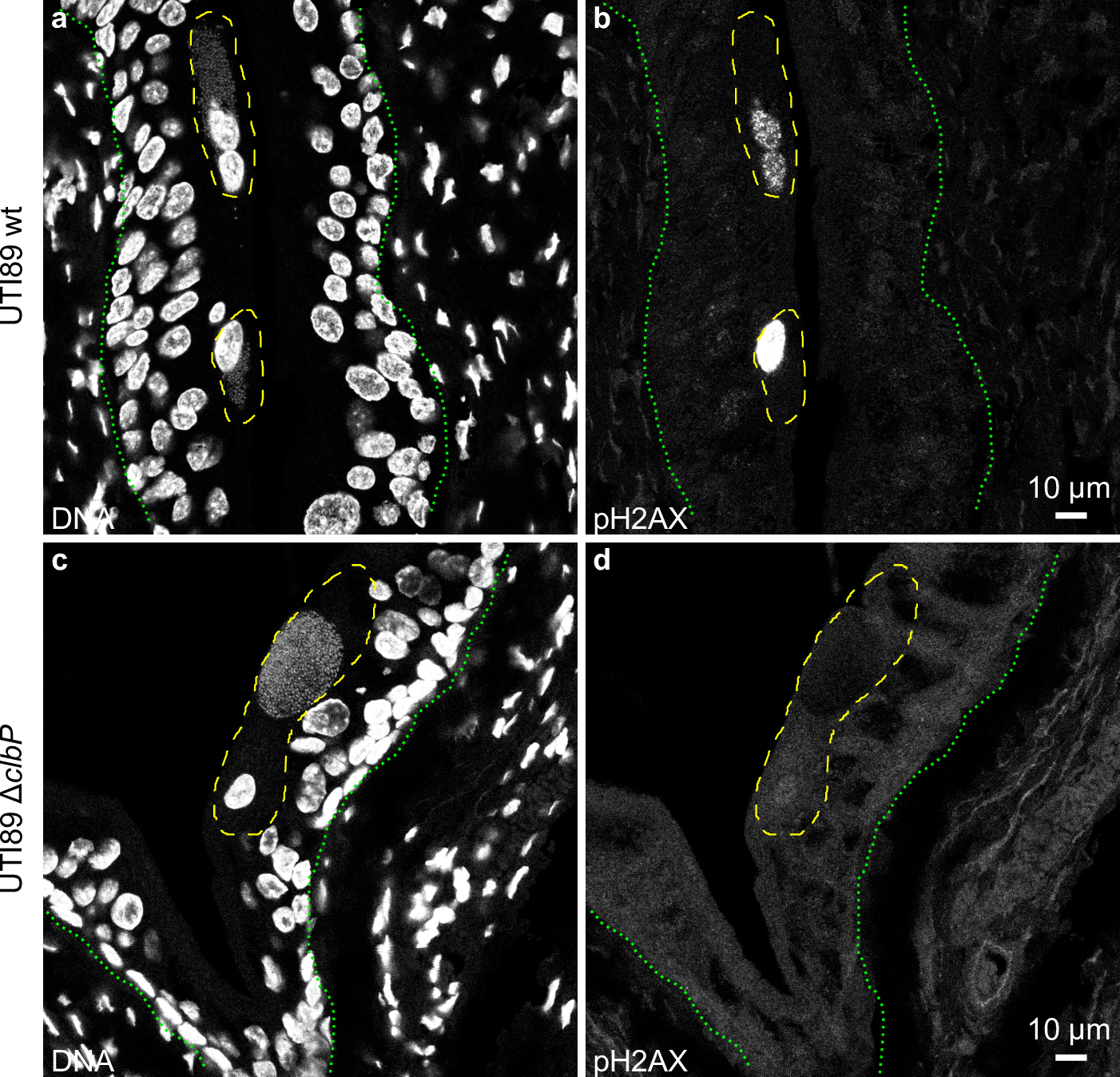

Supplement: S2 Fig — Immunofluorescence staining of pH2AX on paraffin-embedded bladders sections 6 hours post-infection with the wild-type UTI89 (a, b) or the ΔclbP isogenic mutant (c,d). The individual channel images are shown in grayscale. Umbrella cells containing IBCs are circled with yellow dashed line and urothelium is delimited with green dotted line. (TIF) [file ppat.1009310.s002.tif]

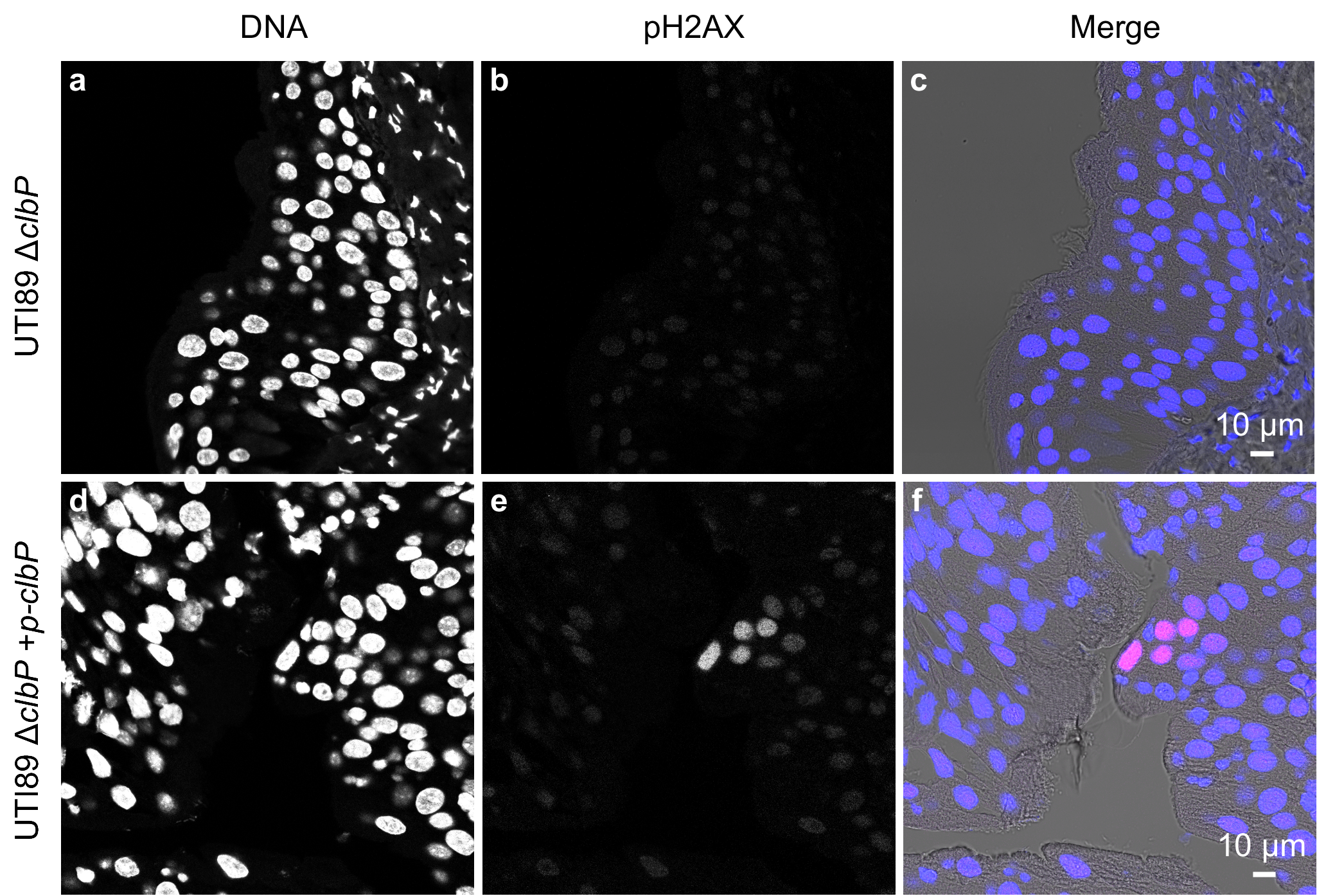

Supplement: S3 Fig — DAPI (DNA: a, d) and immunofluorescence staining of pH2AX (b, e) on paraffin-embedded bladders sections 24 hours post infection by UTI89ΔclbP (a-c) or UTI89ΔclbP+pCM17clbP (d-f). For immunofluorescence, the individual channel images are shown in grayscale. Merged images: blue = DNA, magenta = pH2AX, grey = phase contrast. (TIF) [file ppat.1009310.s003.tif]

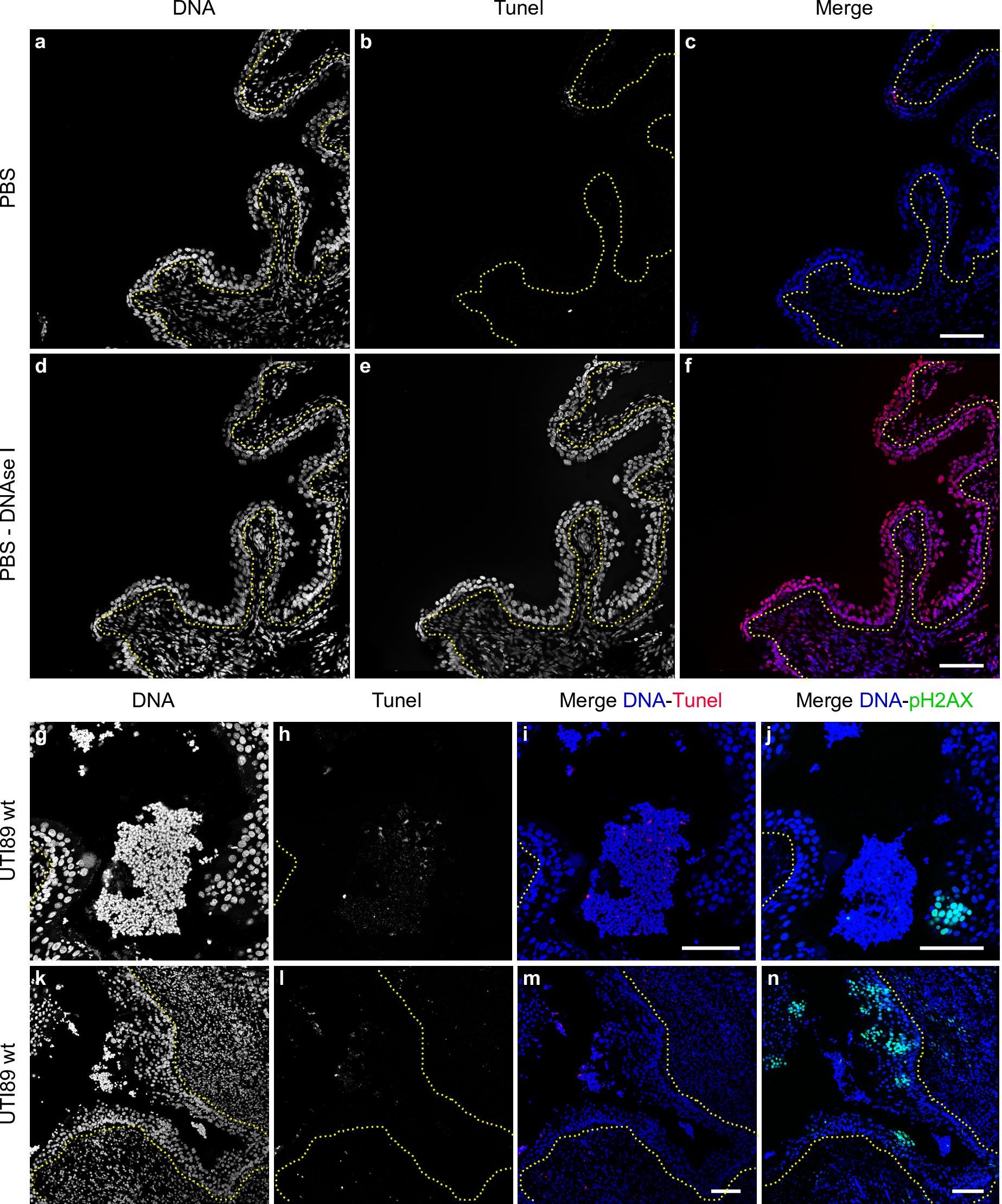

Supplement: S4 Fig — TUNEL staining of paraffine-embedded bladder sections 24 hours post PBS inoculation (a-f) or wild-type UTI89 infection (g-j; k-n) in C3H/HeN mice. Sections were treated with DNAse I as a TUNEL positive control (d-f). TUNEL staining of infected bladder sections was compared to pH2AX immunofluorescence on a serial section (j is a section next to g-i and n is next to k-m). The individual fluorescence channel images are shown in grayscale. Merged images: blue = DNA; red = TUNEL; green = pH2AX. Yellow dotted lines represent the basal membrane of the urothelium. Scale bar = 100 μm. (TIF) [file ppat.1009310.s004.tif]

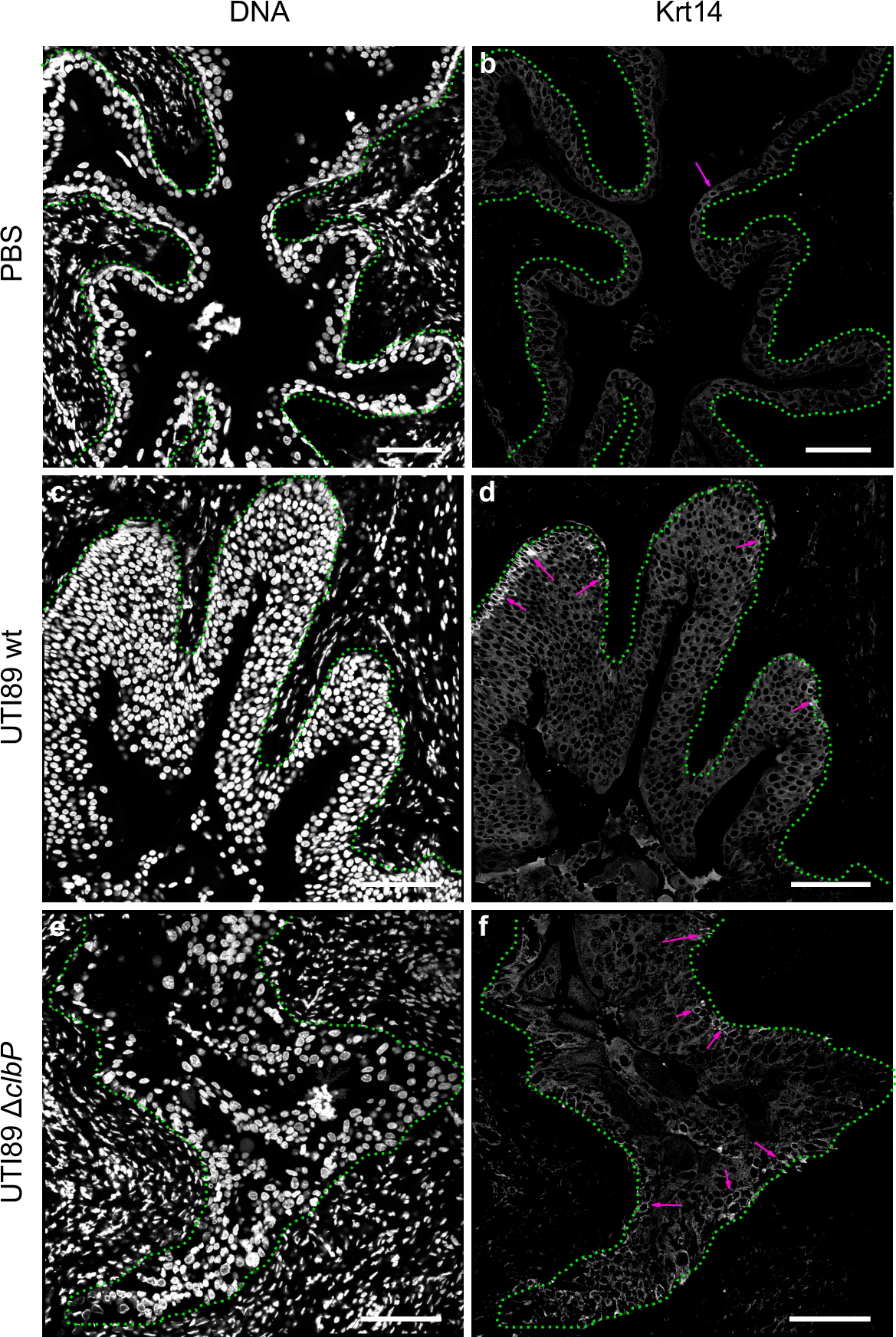

Supplement: S5 Fig — Immunofluorescence staining of Krt14 and DAPI stained DNA on paraffin-embedded bladders sections 24 hours post PBS inoculation (a-b) or infection by UTI89 wild-type (c-d) or UTI89 ΔclbP (e-f). The individual channel images are shown in grayscale. Pink arrows: Areas with Krt14+ cells. Urothelium is delimited with green dotted line. Scale bar = 100 μm. Quantification of Krt14 cells is shown in Fig 5K. (TIF) [file ppat.1009310.s005.tif]

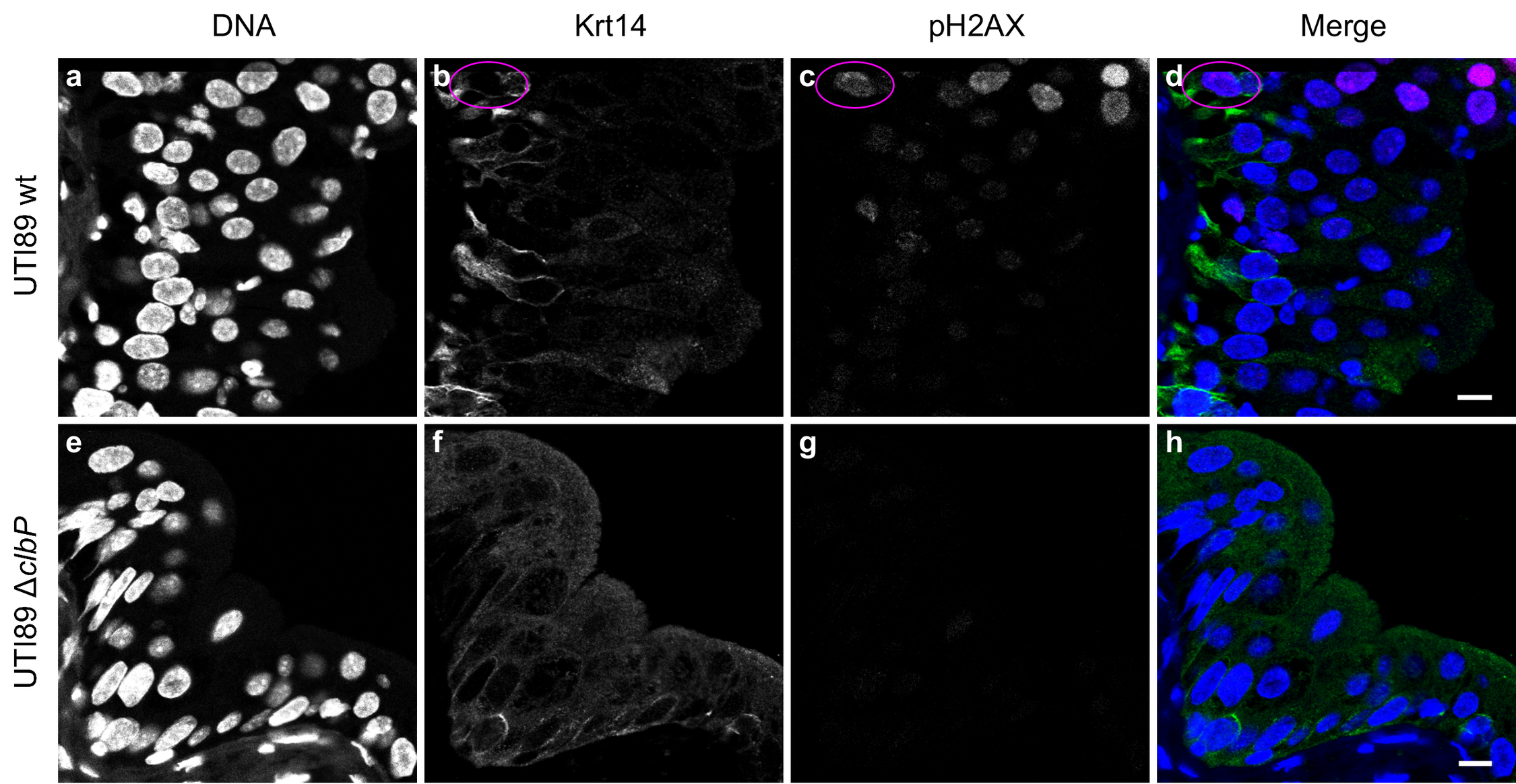

Supplement: S6 Fig — Paraffin-embedded bladders sections were immuno-stained for pH2AX and Krt14 24 hours post infection with UTI89 wild-type (a-d) or UTI89 ΔclbP (e-h). The individual fluorescence channel images are shown in grayscale. Merged images: blue = DNA, magenta = pH2AX, green = Krt14. Cells positive for both Krt14 and pH2AX are circled in pink. Scale bar = 10 μm. (TIF) [file ppat.1009310.s006.tif]

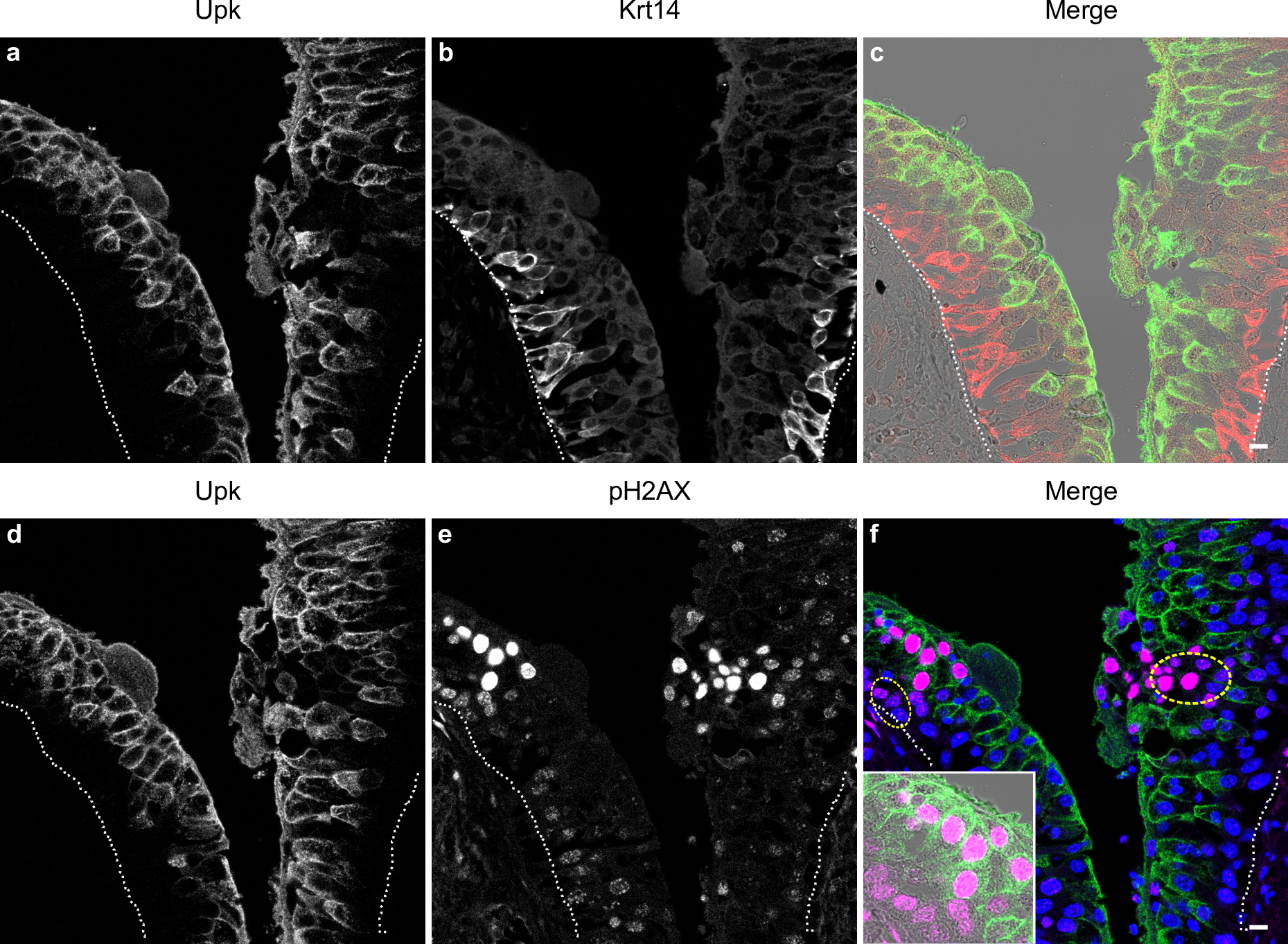

Supplement: S7 Fig — a-c. Immunofluorescence staining of Upk and Krt14 on paraffin-embedded bladder sections 24 hours after UTI89 wild-type infection. d-f. Immunofluorescence staining of Upk and pH2AX on a serial section next to the one shown in a-c. Cells positive for pH2AX but negative for Upk are circled with yellow dashed line. The individual fluorescence channel images are shown in grayscale. Merged images: green = Upk, red = Krt14, magenta = pH2AX, blue = DNA. White dotted lines represent the basal membrane of the urothelium. Scale bar = 10 μm. f. Insert: higher magnification: green = Upk, magenta = pH2AX, gray = contrast. (TIF) [file ppat.1009310.s007.tif]
