# Supplementary material for: Uropathogenic E. coli induces DNA damage in the bladder
Source: PLoS Pathog. 2021 Feb 25;17(2):e1009310. doi: 10.1371/journal.ppat.1009310 (PMC7906301; doi:10.1371/journal.ppat.1009310)
Supplement: S1 Table — Phylogroup, Sequence Types and presence of a pks island were determined from whole genome sequences of UPEC isolates, along with the detection of C14-Asn from the corresponding urine. (DOCX) [file ppat.1009310.s008.docx]

**S1 Table: Human *pks*+ UPEC belong to major lineages of phylogroup B2 ExPEC.**

| Phylogroup | **B2** | | | | | | | **D** | | | **Others** | **Tot.** |
| --- | --- | --- | --- | --- | --- | --- | --- | --- | --- | --- | --- | --- |
| Sequence Type | 73 | 95 | 141 | 404 | 131 | Oth. | Tot. | 69 | Oth. | Tot. | Others |  |
| n | 29 | 27 | 16 | 14 | 23 | 46 | **155** | 26 | 8 | **34** | **36** | 225 |
| Infection |  |  |  |  |  |  |  |  |  |  |  |  |
| Asymptomatic bacteriuria | 4 | 3 | 6 | 0 | 4 | 10 | 27 | 3 | 2 | 5 | 5 | 37 |
| Cystitis | 13 | 2 | 6 | 8 | 13 | 19 | 61 | 6 | 1 | 7 | 16 | 84 |
| Pyelonephritis | 12 | 22 | 4 | 6 | 6 | 17 | 67 | 17 | 5 | 22 | 15 | 104 |
| *pks* island |  |  |  |  |  |  |  |  |  |  |  |  |
| *pks*+ | 29 | 7 | 16 | 14 | 0 | 30 | 96 | 0 | 0 | 0 | 0 | 96 |
| C14-Asn+ | 13 | 6 | 12 | 5 | 0 | 19 | 55 | 0 | 0 | 0 | 0 | 55 |

Phylogroup, Sequence Types and presence of a *pks* island were determined from whole genome sequences of UPEC isolates, along with the detection of C14-Asn from the corresponding urine.
